# A derivative of vitamin B_3_ applied several days after exposure reduces lethality of severely irradiated mice

**DOI:** 10.1038/s41598-021-86870-3

**Published:** 2021-04-12

**Authors:** Aneta Cheda, Ewa M. Nowosielska, Jerzy Gebicki, Andrzej Marcinek, Stefan Chlopicki, Marek K. Janiak

**Affiliations:** 1 Department of Radiobiology and Radiation Protection, Military Institute of Hygiene and Epidemiology, 4 Kozielska St., 01-163 Warsaw, Poland; 2 Institute of Applied Radiation Chemistry, Lodz University of Technology, 15 Wroblewskiego St., 93-590 Lodz, Poland; 3 Jagiellonian Centre for Experimental Therapeutics (JCET), Jagiellonian University, 14 Bobrzynskiego St., 30-348 Kraków, Poland; 4 Chair of Pharmacology, Jagiellonian University Medical College, Jagiellonian University, 16 Grzegorzecka St., 31-531 Kraków, Poland

**Keywords:** Public health, Therapeutics, Chemical biology, Drug discovery

## Abstract

Most, if not all, of the hitherto tested substances exert more or less pronounced pro-survival effects when applied before or immediately after the exposure to high doses of ionizing radiation. In the present study we demonstrate for the first time that 1-methyl nicotinamide (MNA), a derivative of vitamin B_3_, significantly (1.6 to 1.9 times) prolonged survival of BALB/c mice irradiated at LD_30/30_ (6.5 Gy), LD_50/30_ (7.0 Gy) or LD_80/30_ (7.5 Gy) of γ-rays when the MNA administration started as late as 7 days post irradiation. A slightly less efficient and only after the highest dose (7.5 Gy) of γ-rays was another vitamin B_3_ derivative, 1-methyl-3-acetylpyridine (1,3-MAP) (1.4-fold prolonged survival). These pro-survival effects did not seem to be mediated by stimulation of haematopoiesis, but might be related to anti-inflammatory and/or anti-thrombotic properties of the vitamin B_3_ derivatives. Our results show that MNA may represent a prototype of a radioremedial agent capable of mitigating the severity and/or progression of radiation-induced injuries when applied several hours or days after exposure to high doses of ionizing radiation.

## Introduction

During radiological accidents or criminal acts people can be exposed to high (i.e., > 1–2 Gy) doses of ionizing radiation. Short-term exposures at such doses may result in acute radiation syndrome (ARS) and other pathologies whose underlying mechanisms include depression of proliferation of actively dividing progenitor cells, injury to the endothelial lining of blood vessels, inflammation, and thrombosis^1,2^. Such untoward effects could be prevented or alleviated by appropriate radiation countermeasures. Efficacy of the latter in terms of limiting the radiation-induced tissue injury and the ensuing lethality may depend on the time between radiation exposure and administration of a countering agent. Hence, radiation countermeasures can be classified as: (a) radioprotectors, i.e., agents effective when applied before irradiation, (b) radiomitigators, efficient when administered at the time of or soon after radiation exposure, and (c) radioremedial agents, which inhibit progression of radiogenic injuries during the development of the radiation illness^3^.

The quest for an effective radioprotective and, especially, radiotherapeutic agent has a long, but hardly successful, history. In fact, the only radioprotector registered so far is amifostine (Ethyol) which is used to reduce the incidence of xerostomia in patients undergoing radiotherapy for head and neck cancers. However, administration of amifostine is associated with numerous side effects (i.e., nausea, vomiting, hypotension, dizziness, fever, and hypocalcaemia) and it is effective only when applied 15–30 min before the irradiation. Recently though, the US Food and Drug Administration (FDA) has approved three radiomitigators to increase survival of patients with the haematopoietic presentation of ARS all of which are cytokines: granulocyte-colony stimulating factor (G-CSF), polyethylene glycolylated G-CSF, and recombinant granulocyte–macrophage colony stimulating factor (GM-CSF)^4^. However, the search for new and more efficient agents continues and many substances of synthetic and natural origin including cytokines, vitamins, plant extracts, pharmaceuticals, hormones, etc., are being investigated for their potential radioprotective, radiomitigative, and/or radioremedial properties.

The derivatives of vitamin B_3_ such as 1-methylnicotinamide (MNA) demonstrate anti-inflammatory^5^, anti-thrombotic^6^, gastroprotective^7^, hepatoprotective^8,9^, and anti-metastatic effects^10^ mediated by a prostacyclin (PGI_2_)-dependent mechanism. It has been also reported that derivatives of vitamin B_3_ reduce the levels of pro-inflammatory cytokines such as IL-1β, IL-6, IL-8, and TNF-α in the blood and have perform vasoprotective functions^9,11,12^. It is likely, therefore, that such compounds may prove effective in the prevention and treatment of radiation-induced pathologies. Moreover, since MNA at concentrations used in the present investigation is a stable and non-toxic molecule^5,6^, it’s application is unlikely to evoke serious side effects. So far, however, radioprotective, radiomitigative, or radioremedial capacities of MNA and other derivatives of vitamin B_3_ have not been investigated. Therefore, in the present studies we aimed to assess the effects of the selected derivatives of vitamin B_3_ on the survival of mice exposed to LD_30/30_, LD_50/30_ or LD_80/30_ of γ radiation and tried to discern possible mechanisms involved.

## Results

As determined by us in a pilot study the 30-day mortality rates of the relatively radiosensitive 6- to 8-week-old BALB/c mice averaged 31.5% after the whole-body irradiation (WBI) of the animals at 6.5 Gy, 49.8%—after WBI at 7.0 Gy, and 82.3%—after WBI at 7.5 Gy of γ-rays. Hence, the doses used in this study were regarded as LD_30/30_, LD_50/30_, and LD_80/30_, respectively.

**Figure 1:**
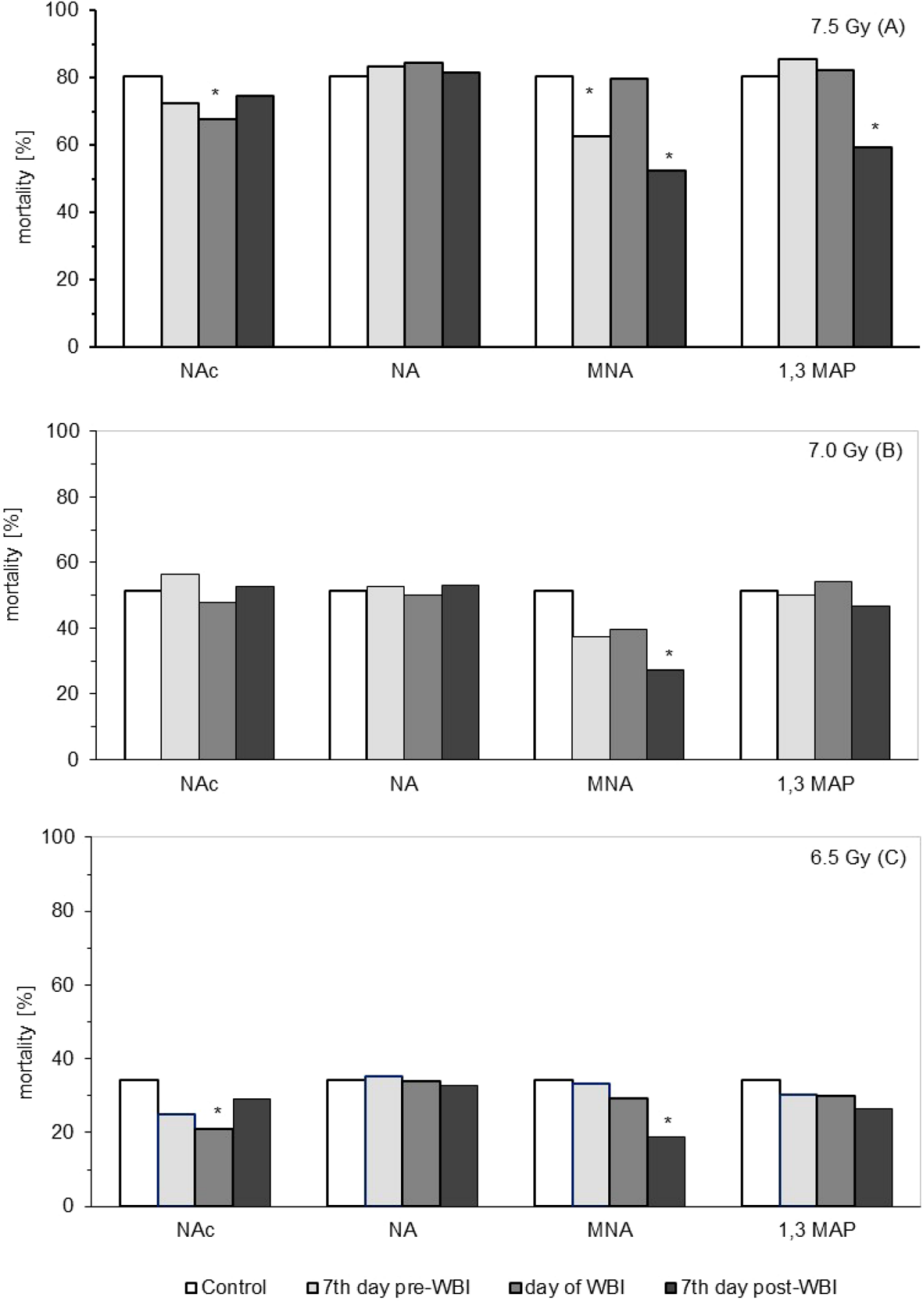
Mortality (expressed as survival probability) of BALB/c mice at the end of the 30-day survival assay after exposure to WBI at 7.5 (**A**), 7.0 (**B**), or 6.5 (**C**) Gy of γ-rays. Mean values obtained from experiments conducted on 24 animals per group are presented; NAc—nicotinic acid; NA—nicotinamide; MNA—1-methylnicotinamide; 1,3-MAP –1-methyl-3-acetylpyridine; Control—mice exposed to WBI at 6.5, 7.0, or 7.5 Gy γ-rays; 7th day pre-WBI—mice exposed to WBI at 6.5, 7.0, or 7.5 Gy γ-rays and fed the vitamin B_3_ derivatives from the 7th day before WBI; day of WBI—mice exposed to WBI at 6.5, 7.0, or 7.5 Gy γ-rays and fed the vitamin B_3_ derivatives from the day of WBI; 7th day post-WBI—mice exposed to WBI at 6.5, 7.0, or 7.5 Gy γ-rays and fed the vitamin B_3_ derivatives from the 7th day after WBI. *Indicates statistically significant (*p* < 0.05) difference from the results obtained in the irradiated control group.

To determine the presumable radioprotective, radiomitigative and/or radioremedial potentials of the selected derivatives of vitamin B_3_ BALB/c mice were fed these compounds from day 7 before, the day of, or day 7 after WBI of the animals at 6.5, 7.0 or 7.5 Gy of γ-rays and their survival was assessed over 30 days post-exposure. As indicated in Fig. 1, mortality rate at the end of the 30-day survival assay after WBI at 7.5 Gy was markedly decreased when the animals were given the following compounds in drinking water: a) 1-methylnicotinamide (MNA) beginning from the 7th day before or after WBI, b) nicotinic acid (NAc) from the day of WBI, and c) 1-methyl, 3-acetylpyridine (1,3 MAP) from the 7th day after WBI (Fig. 1A). Comparably effective was administration of MNA from the 7th day after WBI at 7.0 and 6.5 Gy and of NAc from the day of the irradiation at 6.5 Gy of γ-rays (Fig. 1B, C). Administration of nicotinamide (NA) did not seem to affect the mortality of the mice exposed to any of the radiation doses.

**Figure 2:**
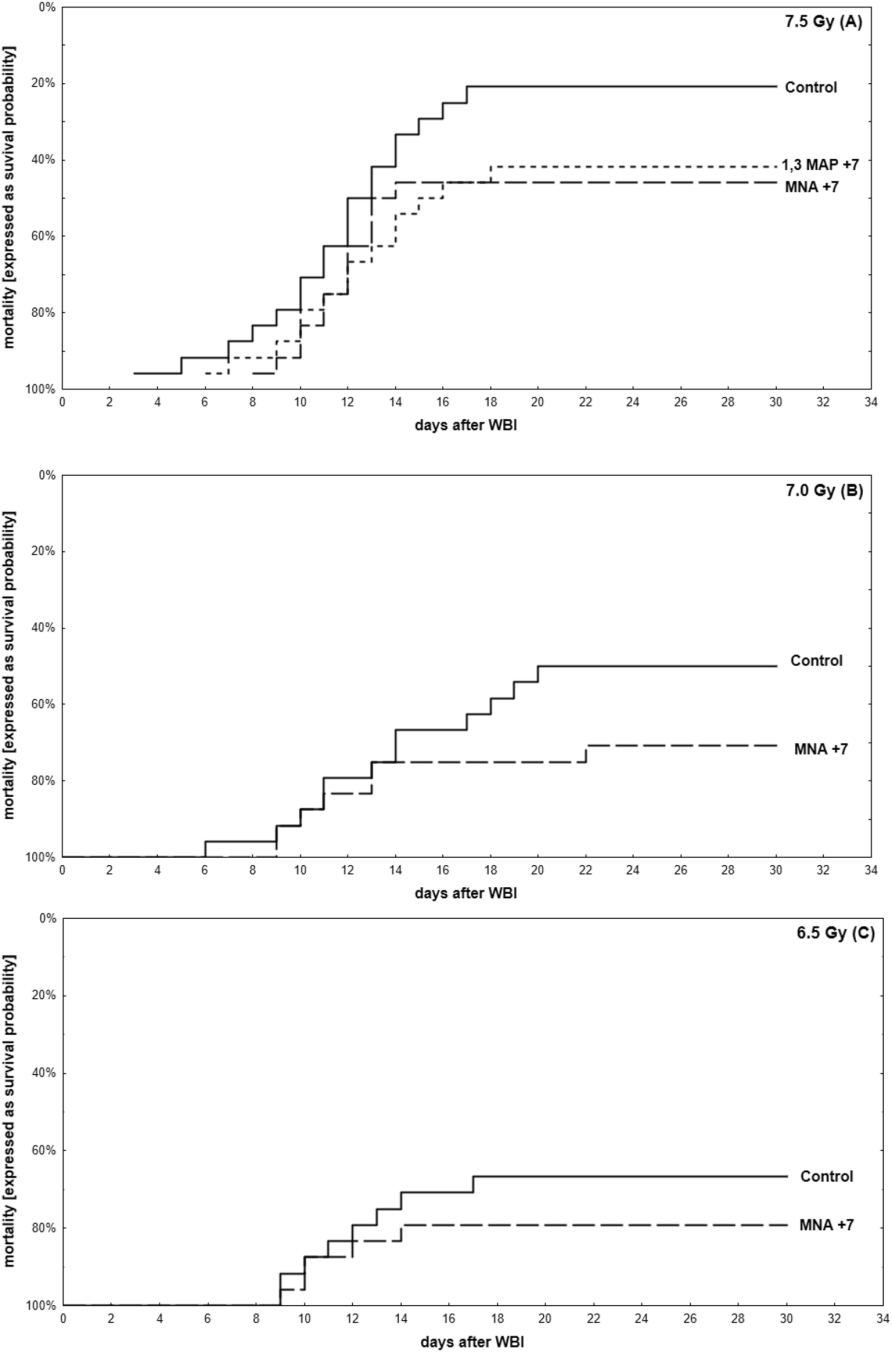
Mortality (expressed as survival probability) of BALB/c mice exposed to WBI at 7.5 (**A**), 7.0 (**B**), or 6.5 (**C**) Gy of γ-rays and fed the vitamin B_3_ derivatives in drinking water (100 mg/kg b.m./day) from the 7th day after WBI. Only results significantly different from the control group are presented. Mean values obtained from experiments conducted on 24 animals per group are presented; Control—mice exposed to WBI at 6.5, 7.0, or 7.5 Gy γ-rays; MNA + 7—mice fed 1-methylnicotinamide (MNA) from the 7th day after WBI at 6.5, 7.0, or 7.5 Gy γ-rays; 1,3 MAP + 7—mice fed 1-methyl-3-acetylpyridine (1,3-MAP) from the 7th day after WBI at 7.5 Gy γ-rays.

Radioremedial effects, i.e. prolongation of survival when the tested compounds started to be applied 7 days after the irradiation, were clearly demonstrable only for MNA and 1,3 MAP. As shown in Fig. 2A, when mice were exposed to 7.5 Gy of γ-rays the radioremedial effects of the two compounds were comparable: mortality decreased from about 81% to about 53% and 59% in case of MNA and MAP, respectively. Surprisingly, only the former compound exhibited similar effects in mice exposed to 7.0 and 6.5 Gy of radiation (Fig. 2A–C) Notably, on the 30th day of observation the surviving mice were still in the relatively good condition and did not seem to be moribund (Supplementary Fig. 1 and Table 1). This observation indicated that the treatment was effective and sustainable.

**Table 1:** Bone marrow, spleen, and blood cell counts in BALB/c mice exposed to WBI at 6.5, 7.0 or 7.5 Gy γ-rays and in sham-exposed mice.

Day after exposure	Parameters		0 Gy	6.5 Gy	7.0 Gy	7.5 Gy
Day 7	Cellularity	BM × 10^5^	663.8 ± 59.1	15.5 ± 1.9*	8.3 ± 1.1*	4.3 ± 0.4*
		Spleen × 10^6^	180.0 ± 11.2	5.1 ± 0.9*	4.7 ± 0.3*	1.5 ± 0.2*
	Cells/ml	WBC × 10^3^	9.0 ± 3.8	2.0 ± 1.1*	1.3 ± 0.5*	0.7 ± 0.4*
		PLT × 10^3^	665.5 ± 285.8	148.0 ± 78.9*	71.0 ± 29.2*	57.1 ± 17.9*
		RBC × 10^6^	9.0 ± 1.,8	8.2 ± 1.2*	7.8 ± 1.1*	7.4 ± 0.8*
Day 10	Cellularity	BM × 10^5^	663.8 ± 59.1	10.2 ± 0.9*	6.9 ± 0.5*	2.9 ± 0.2*
		Spleen × 10^6^	180.0 ± 11.2	4.2 ± 0.3*	1.9 ± 0.2*	0.9 ± 0.1*
	Cells/ml	WBC × 10^3^	9.0 ± 3.8	1.2 ± 0.7*	0.7 ± 0.5*	0.4 ± 0.2*
		PLT × 10^3^	665.5 ± 285,8	205.5 ± 38,2*	114.0 ± 31.2*	67.5 ± 21.3*
		RBC × 10^6^	9.0 ± 2,8	6.3 ± 1,0*	5.8 ± 0,8*	5.7 ± 0.7*
Day 14	Cellularity	BM × 10^5^	663.8 ± 59.1	38.3 ± 2.9*	18.3 ± 1.0*	6.3 ± 0.2*
		Spleen × 10^6^	180.0 ± 11.2	8.3 ± 1.4*	5.0 ± 0.6*	3.7 ± 0.1*
	Cells/ml	WBC × 10^3^	9.0 ± 3.8	0.8 ± 0.5*	0.4 ± 0.2*	0.2 ± 0.1*
		PLT × 10^3^	665.5 ± 285,8	111.0 ± 48.2*	66.5 ± 23.7*	40.5 ± 18.2*
		RBC × 10^6^	9.0 ± 2,8	7.1 ± 1.1*	7.5 ± 1.0*	6.4 ± 0.7*
Day 30	Cellularity	BM × 10^5^	663.8 ± 59.1	590.0 ± 68.5	582.1 ± 71.8	522.7 ± 67.1
		Spleen × 10^6^	180.0 ± 17.2	168.6 ± 15.3	155.2 ± 14.9	128.4 ± 11.9
	Cells/ml	WBC × 10^3^	9.0 ± 3.8	9.2 ± 2.5	9.1 ± 1.9	7.8 ± 1.8
		PLT × 10^3^	665.5 ± 285.8	463.0 ± 135.0*	445.5 ± 154.7*	363.0 ± 114.5*
		RBC × 10^6^	9.0 ± 2.8	8.2 ± 0.9	8.2 ± 0.9	8.5 ± 0.9

Mean values ± SD obtained from experiments conducted on 20 mice per group are presented. *BM*—bone marrow cells, Spleen—spleen cells, WBC—white blood cells; PLT—platelets; RBC—red blood cells; 0 Gy—sham-exposed, control mice; 6.5 Gy—mice exposed to WBI at 6.5 Gy γ-rays; 7.0 Gy—mice exposed to WBI at 7.0 Gy γ-rays; 7.5 Gy—mice exposed to WBI at 7.0 Gy γ-rays. Day 7—7th day after WBI at 6.5, 7.0 or 7.5 Gy γ-rays; Day 10—10th day after WBI at 6.5, 7.0 or 7.5 Gy γ-rays; Day 14—14th day after WBI at 6.5, 7.0 or 7.5 Gy γ-rays; Day 30—30th day after WBI at 6.5, 7.0 or 7.5 Gy γ-rays. Since the results at 0 Gy for days 7, 10, 14 and 30 were similar they have been averaged.

*Indicates statistically significant (*p* < 0.05) difference from the results obtained in the sham-exposed group.

As shown in Table 1 total numbers of bone marrow and spleen cells were dramatically, but proportionally to the dose, reduced after single WBI of mice at 6.5, 7.0, or 7.5 Gy γ-rays (Table 1). The reduction was detectable between the 7th and 14th days post-irradiation, but on the 30th day the bone marrow and spleen cellularities recovered almost to the level observed in the sham-exposed, control mice. Application of the tested vitamin B_3_ derivatives did not significantly affect the numbers of bone marrow and spleen cells as compared to the numbers counted in the irradiated mice which drank pure water (Supplementary Table 2). Likewise, acute WBI of mice at 6.5, 7.0, or 7.5 Gy γ-rays dramatically, but proportionally to the dose, reduced the numbers of circulating leukocytes (WBC), platelets (PLT), and red blood cells (RBC) until the 14th day after the irradiation (Table 1). These blood cell counts also tended to return to the baseline levels on the 30th day post-exposure. Compared to the period between the 7th and 14th days after the irradiation, the number of PLT increased significantly on the 30th day but was still lower than in the sham-exposed mice. When the irradiated mice were fed each of the tested compounds no significant changes in the numbers of WBC, PLT, and RBC cells were detected compared to the similarly irradiated mice which were not given these compounds (Supplementary Table 2). Similar results were obtained for the levels of haemoglobin and haematocrit (Supplementary Table 2).

We next estimated changes in the production of the selected pro-inflammatory cytokines in mice exposed to γ-rays and fed the tested derivatives of vitamin B_3_. For these experiments the mice were exposed only at 6.5 Gy, since almost 70% of the animals survived such irradiation until day 30 and we were able to obtain the required amount of blood for the analyses.

**Figure 3:**
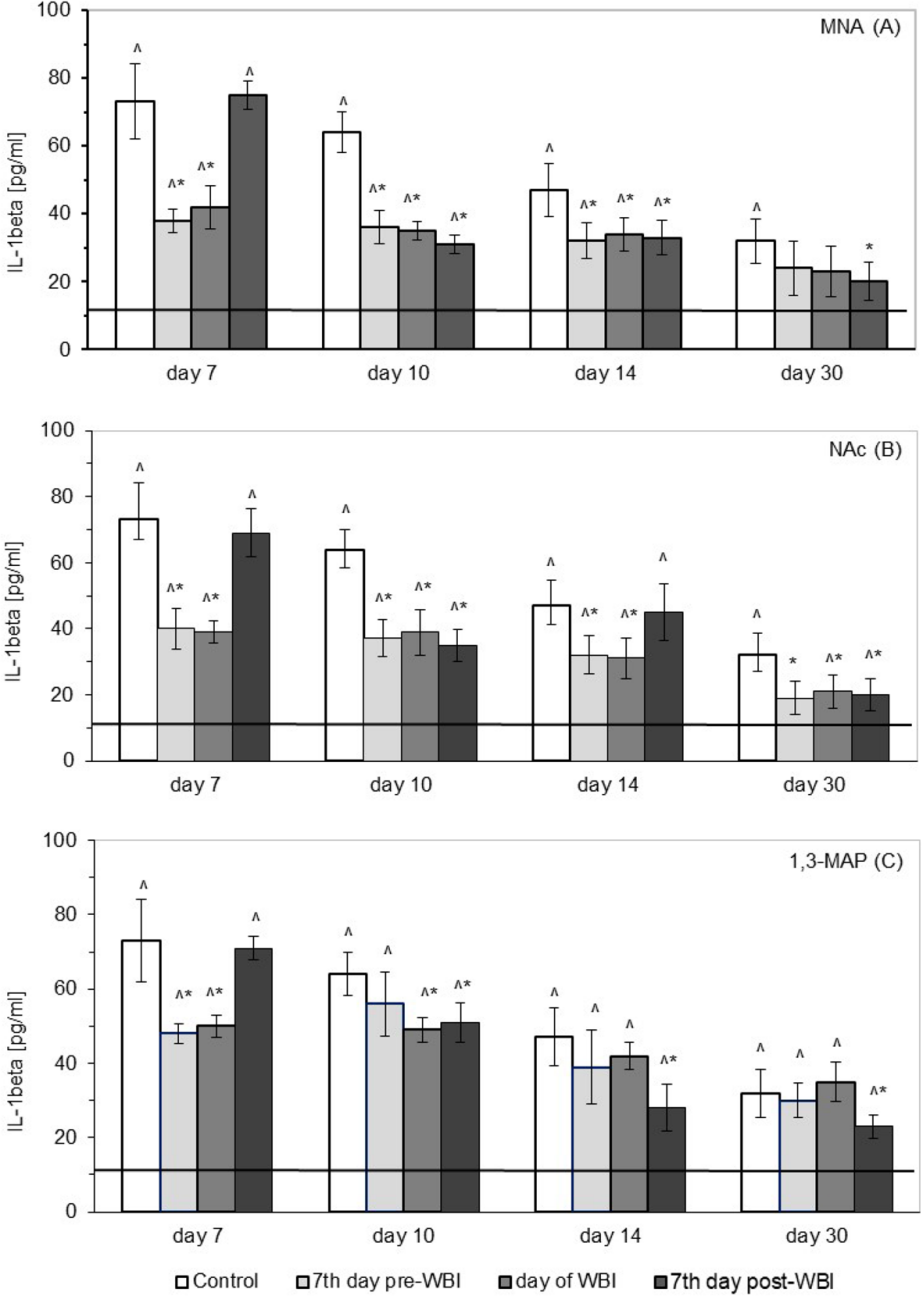
Serum levels of interleukin-1β (IL-1β) [pg/ml] in BALB/c mice exposed to WBI at 6.5 Gy γ-rays and fed: MNA—1-methylnicotinamide (**A**), NAc—nicotinic acid (**B**), or 1,3-MAP—1-methyl-3-acetylpyridine (**C**) in drinking water (100 mg/kg b.m./day) starting 7 days before, on the day of, or 7 days after WBI. Mean values ± SD obtained from experiments conducted on 20 mice per group are presented. Day 7—7th day after WBI at 6.5 Gy γ-rays; Day 10—10th day after WBI at 6.5 Gy γ-rays; Day 14—14th day after WBI at 6.5 Gy γ-rays; Day 30—30th day after WBI at 6.5 Gy γ-rays; Control—mice exposed to WBI at 6.5 Gy γ-rays; 7th day pre-WBI—mice exposed to WBI at 6.5 Gy γ-rays and fed the vitamin B_3_ derivatives from the 7th day before WBI; day of WBI—mice exposed to WBI at 6.5 Gy γ-rays and fed the vitamin B_3_ derivatives from the day of WBI; 7th day post-WBI—mice exposed to WBI at 6.5 Gy γ-rays and fed the vitamin B_3_ derivatives from the 7th day after WBI. The solid line represents the serum level of IL-1β in the sham-irradiated mice. *Indicates statistically significant (*p* < 0.05) difference from the results obtained in the irradiated control group. **^**indicates statistically significant (*p* < 0.05) difference from the results obtained in the sham-irradiated mice.

As shown in Fig. 3 the level of interleukin-1β (IL-1β) in the serum significantly increased after WBI at 6.5 Gy compared to the level observed in the sham-irradiated mice (solid line). The most pronounced effect was detected on the 7th day post WBI after which the concentration of IL-1β slowly declined, but on the 30th day was still about 2.5-fold higher than in the sham-irradiated mice (Fig. 3). The radiation-enhanced production of IL-1β was significantly down-regulated by administration of MNA from day 7 before or from the day of WBI, the effect being detectable between the 7th and 14th days after WBI (Fig. 3A). Similar reduction was seen when MNA started to be applied on the 7th day post irradiation, but this time the effect was detected between days 10 and 30, but not on day 7 after WBI (Fig. 3A). Pronounced decrease in the up-regulated levels of IL-1β was also detectable throughout the observation period when the irradiated mice were given NAc from day 7 prior to or from the day of WBI. In contrast, administration of NAc from day 7 after WBI suppressed the increased production of IL-1β on the 10th and 30th day, but not on the 7th and 14th day post WBI (Fig. 3B). Significant reductions of the up-regulated IL-1β were also seen in mice fed 1,3-MAP and the effect depended on the time of the application of the compound: when the application started 7 days before WBI the effect was seen only on the 7th day post irradiation, when the application started on the day of WBI the reduction was detected 7 and 10 days post exposure, and when the compound began to be applied 7 days after WBI the decreased levels of IL-1β were seen between days 10 and 30, but not on the 7th day post irradiation (Fig. 3C).

**Figure 4:**
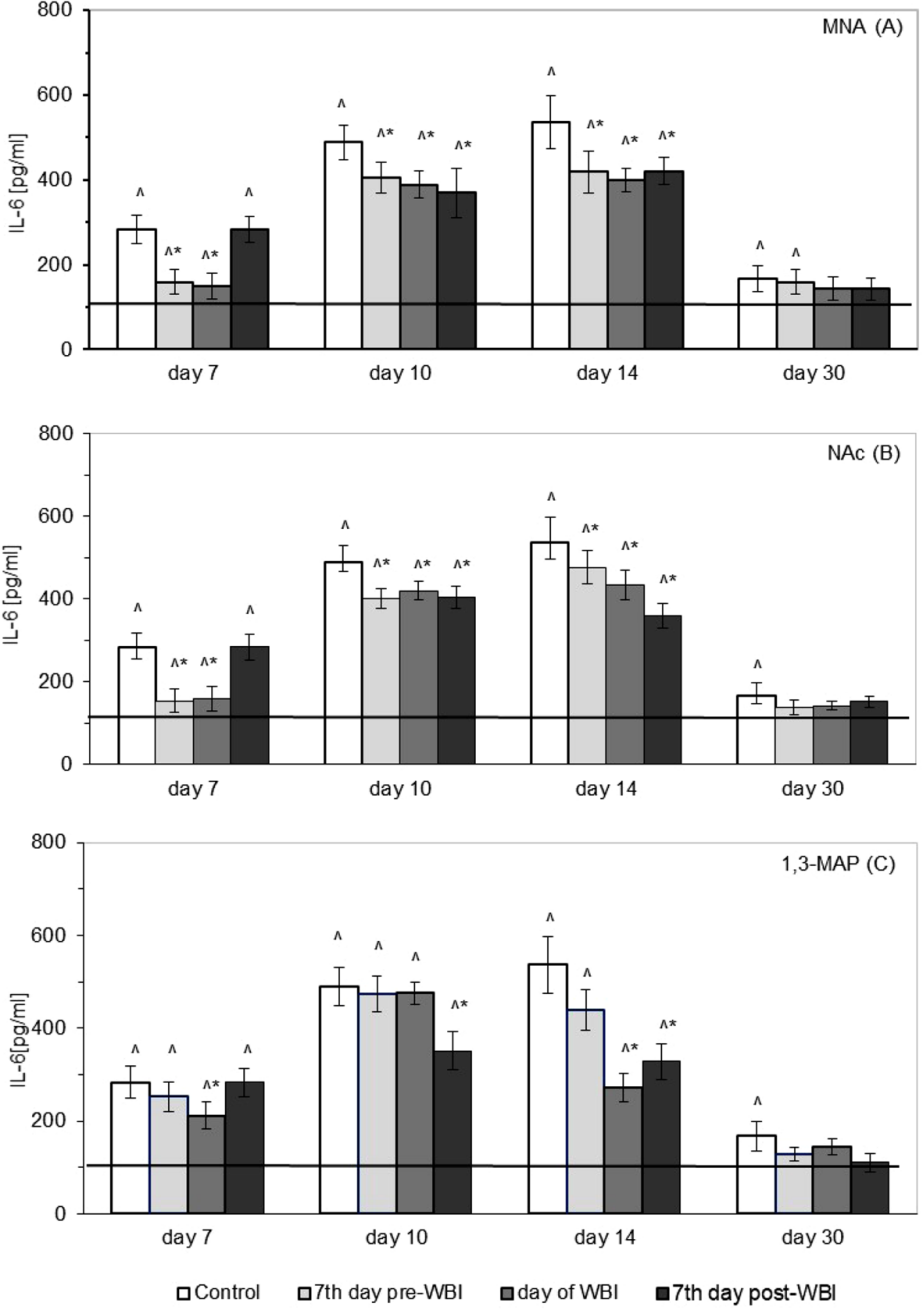
Serum levels of interleukin 6 (IL-6) [pg/ml] in BALB/c mice exposed to WBI at 6.5 Gy γ-rays and fed: MNA—1-methylnicotinamide (**A**), NAc—nicotinic acid (**B**), or 1,3-MAP—1-methyl-3-acetylpyridine (**C**) in drinking water (100 mg/kg b.m./day) starting 7 days before, on the day of, or 7 days after WBI. Mean values ± SD obtained from experiments conducted on 20 mice per group are presented. Day 7—7th day after WBI at 6.5 Gy γ-rays; Day 10—10th day after WBI at 6.5 Gy γ-rays; Day 14—14th day after WBI at 6.5 Gy γ-rays; Day 30—30th day after WBI at 6.5 Gy γ-rays; Control—mice exposed to WBI at 6.5 Gy γ-rays; 7th day pre-WBI—mice exposed to WBI at 6.5 Gy γ-rays and fed the vitamin B_3_ derivatives from the 7th day before WBI; day of WBI—mice exposed to WBI at 6.5 Gy γ-rays and fed the vitamin B_3_ derivatives from the day of WBI; 7th day post-WBI—mice exposed to WBI at 6.5 Gy γ-rays and fed the vitamin B_3_ derivatives from the 7th day after WBI. The solid line represents the serum level of IL-6 in the sham-irradiated mice. *Indicates statistically significant (*p* < 0.05) difference from the results obtained in the irradiated control group. ^indicates statistically significant (*p* < 0.05) difference from the results obtained in the sham-irradiated mice.

Figure 4 demonstrates that irradiation of mice at 6.5 Gy led to the significant up-regulation in the serum level of IL-6 compared to the level observed in the sham-irradiated mice (solid line). The most pronounced effect was detected on the 10th and 14th days after WBI after which the concentration of the cytokine declined, but on the 30th day was still markedly higher than in the sham-exposed mice (Fig. 4). Administration of MNA from day 7 prior to or the day of WBI significantly diminished the radiation-induced production of IL-6, as noted between the 7th and 14th days after WBI, while administration of this compound from day 7 after WBI led to the significant decrease in the level of IL-6 on the 10th and 14th, but not on the 7th day post exposure (Fig. 4A). The marked down-regulation of the elevated level of IL-6 was also detected between the 7th and 14th days after single WBI when mice were fed NAc from day 7 prior to or the day of WBI, whereas administration of NAc from day 7 after WBI led to the significantly reduced production of IL-6 on the 10th and 14th day after WBI (Fig. 4B). On the 7th and 14th, but not the 10th day after WBI the production of IL-6 was also markedly supressed by administration of 1,3-MAP from the day of WBI, whereas application of this compound from day 7 post-WBI led to a significant reduction of the IL-6 level noted on the 10th and 14th days after WBI (Fig. 4C). In all but one of the examined groups of mice treated with MNA, NAc, and 1,3-MAP the level of IL-6 in the serum returned on day 30 of observation almost to the baseline level, the only exception being mice fed MNA from day 7 prior to the irradiation in which the level of this cytokine was still markedly elevated.

**Figure 5:**
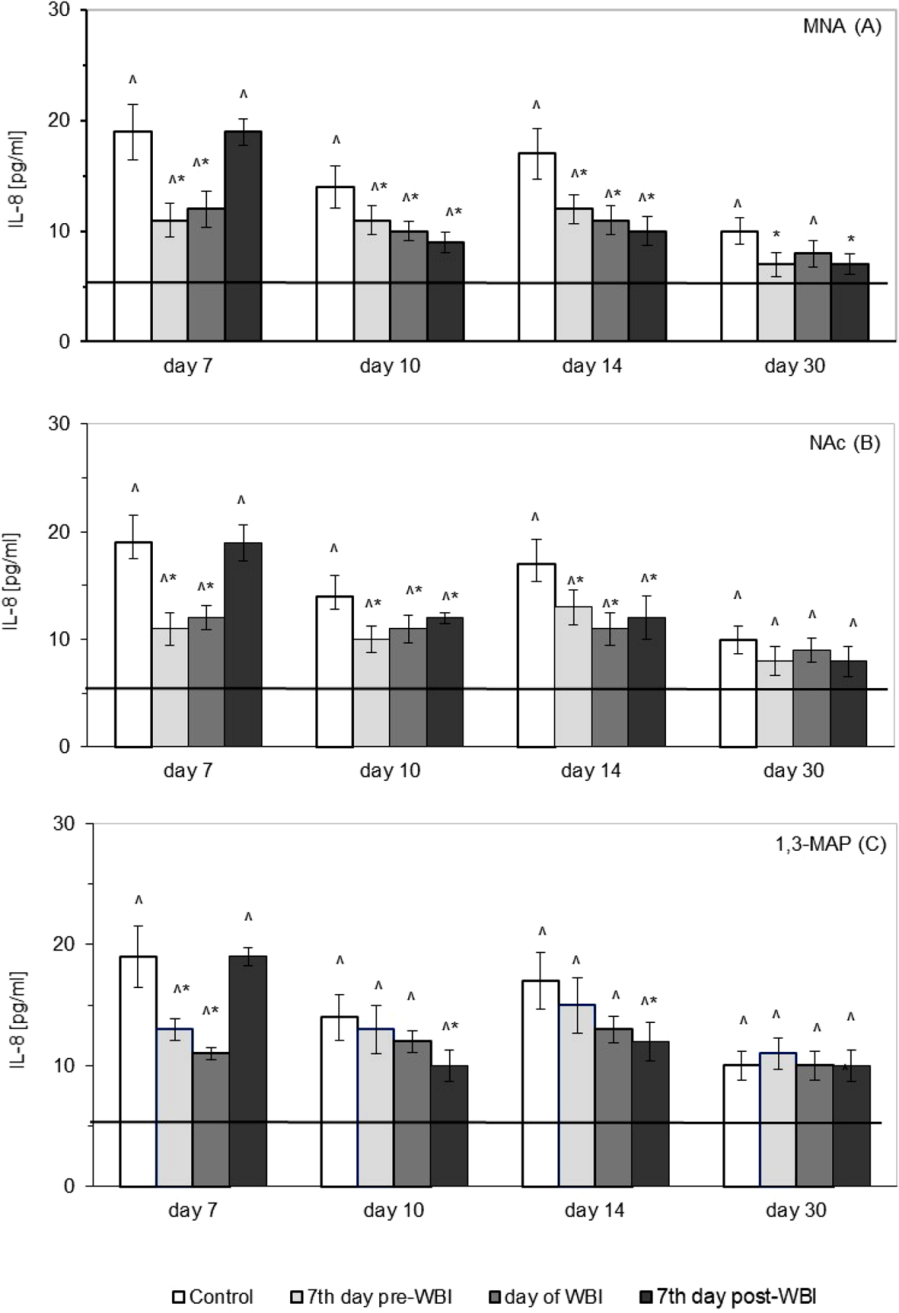
Serum levels of interleukin 8 (IL-8) [pg/ml] in BALB/c mice exposed to WBI at 6.5 Gy γ-rays and fed MNA—1-methylnicotinamide (**A**), NAc—nicotinic acid (**B**), or 1,3-MAP—1-methyl-3-acetylpyridine (**C**) in drinking water (100 mg/kg b.m./day) starting 7 days before, on the day of, or 7 days after WBI. Mean values ± SD obtained from experiments conducted on 20 mice per group are presented. Day 7—7th day after WBI at 6.5 Gy γ-rays; Day 10—10th day after WBI at 6.5 Gy γ-rays; Day 14—14th day after WBI at 6.5 Gy γ-rays; Day 30—30th day after WBI at 6.5 Gy γ-rays; Control—mice exposed to WBI at 6.5 Gy γ-rays; 7th day pre-WBI—mice exposed to WBI at 6.5 Gy γ-rays and fed the vitamin B_3_ derivatives from the 7th day before WBI; day of WBI—mice exposed to WBI at 6.5 Gy γ-rays and fed the vitamin B_3_ derivatives from the day of WBI; 7th day post-WBI—mice exposed to WBI at 6.5 Gy γ-rays and fed the vitamin B_3_ derivatives from the 7th day after WBI. The solid line represents the serum level of IL-8 in the sham-irradiated mice. *Indicates statistically significant (*p* < 0.05) difference from the results obtained in the irradiated control group. ^indicates statistically significant (*p* < 0.05) difference from the results obtained in the sham-irradiated mice.

As indicated in Fig. 5 serum levels of IL-8 significantly increased after WBI of mice at 6.5 Gy, as compared to the level of the cytokine observed in the sham-irradiated animals (solid line). The effect was most pronounced on the 7th day post-WBI after which the concentration of IL-8 slightly declined on the 10th day, rose again on the 14th day and then declined once more, but on the 30th day it was still almost twice as high as in the sham-irradiated mice (Fig. 5). The radiation-enhanced production of IL-8 was significantly reduced by administration of MNA from day 7 prior to WBI, the effect being detectable between the 7th and 30th days after WBI. Similar reduction was seen when MNA started to be applied on the day of WBI, but this time the effect was detected between days 7 and 14, but not on day 30 post irradiation. In contrast, administration of MNA from day 7 after WBI led to the significant decrease in the level of IL-8 detectable between the 10th and 30th day of the observation (Fig. 5A). Pronounced reduction in the up-regulated levels of IL-8 were also observed between the 7th and 14th day after WBI when mice were fed NAc from day 7 prior to and the day of WBI, whereas administration of NAc from day 7 after WBI significantly down-regulated production of IL-8 only on the 10th and 14th day after WBI (Fig. 5B). In turn, administration of 1,3-MAP from day 7 prior to or the day of WBI resulted in the significant decrease in the level of IL-8 only on the 7th day after WBI, while application of the compound from day 7 post-WBI markedly suppressed the level of this cytokine on the 10th and 14th day after WBI (Fig. 5C).

**Figure 6:**
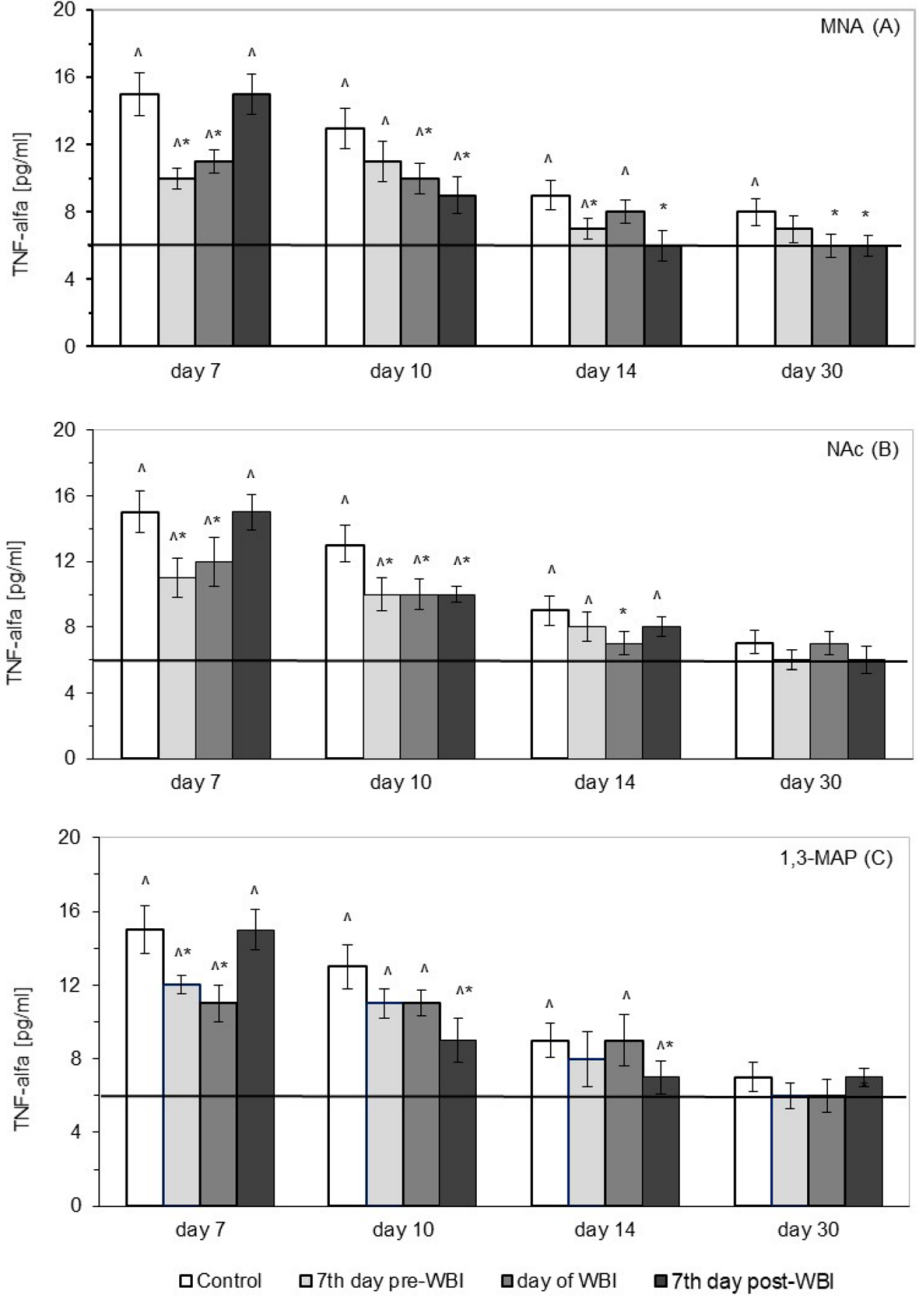
Serum levels of tumour necrosis factor α (TNF-α) [pg/ml] in BALB/c mice exposed to WBI at 6.5 Gy γ-rays and fed MNA—1-methylnicotinamide (**A**), NAc—nicotinic acid (**B**), or 1,3-MAP—1-methyl-3-acetylpyridine (**C**) in drinking water (100 mg/kg b.m./day) starting 7 days before, on the day of, or 7 days after WBI. Mean values ± SD obtained from experiments conducted on 20 mice per group are presented. Day 7—7th day after WBI at 6.5 Gy γ-rays; Day 10—10th day after WBI at 6.5 Gy γ-rays; Day 14—14th day after WBI at 6.5 Gy γ-rays; Day 30—30th day after WBI at 6.5 Gy γ-rays; Control—mice exposed to WBI at 6.5 Gy γ-rays; 7th day pre-WBI—mice exposed to WBI at 6.5 Gy γ-rays and fed the vitamin B_3_ derivatives from the 7th day before WBI; day of WBI—mice exposed to WBI at 6.5 Gy γ-rays and fed the vitamin B_3_ derivatives from the day of WBI; 7th day post-WBI—mice exposed to WBI at 6.5 Gy γ-rays and fed the vitamin B_3_ derivatives from the 7th day after WBI. The solid line represents the serum level of TNF-α in the sham-irradiated mice. *Indicates statistically significant (*p* < 0.05) difference from the results obtained in irradiated control group. ^indicates statistically significant (*p* < 0.05) difference from the results obtained in the sham-irradiated mice.

Similar to the post-irradiation increases in the levels of IL-1β, IL-6, and IL-8, serum concentration of tumour necrosis factor-α (TNF-α) was significantly up-regulated after WBI of the mice at 6.5 Gy (Fig. 6). The effect was most pronounced on the 7th day after WBI, after which the level of this cytokine declined and on the 30th day was almost similar to that found in the sham-irradiated mice. The radiation-induced production of TNF-α was significantly reduced by administration of MNA from day 7 prior to WBI, the effect being detectable on the 7th and 14th day after WBI. Similar reduction was seen when MNA started to be applied on the day of WBI, but this time the effect was detected on the 7th, 10th, and 30th day after WBI, while administration of MNA from day 7 after WBI led to the significant decline in the level of TNF-α between days 10 and 30 (Fig. 6A). Markedly decreased levels of TNF-α were also detected on the 7th and 10th day after WBI, when mice were fed NAc from day 7 prior to WBI. When administration of NAc started on the day of WBI the significantly suppressed production of TNF-α was observed between days 7 and 14 after WBI, whereas application of NAc from day 7 after WBI resulted in the significantly reduced production of TNF-α only on the 10th day after WBI (Fig. 6B). Pronounced decrease in the up-regulated levels of TNF-α was detectable only on the 7th day after WBI, when the irradiated mice were started to be given 1,3-MAP from day 7 prior to and the day of WBI, whereas administration of the compound from day 7 post WBI markedly reduced the level of TNF-α on the 10th and 14th day after WBI (Fig. 6C).

Irrespectively of the time of its application, administration of NA to the irradiated mice did not affect the elevated levels of IL-1β, IL-6, IL-8, and TNF-α in these animals (Supplementary Fig. 2).

**Figure 7:**
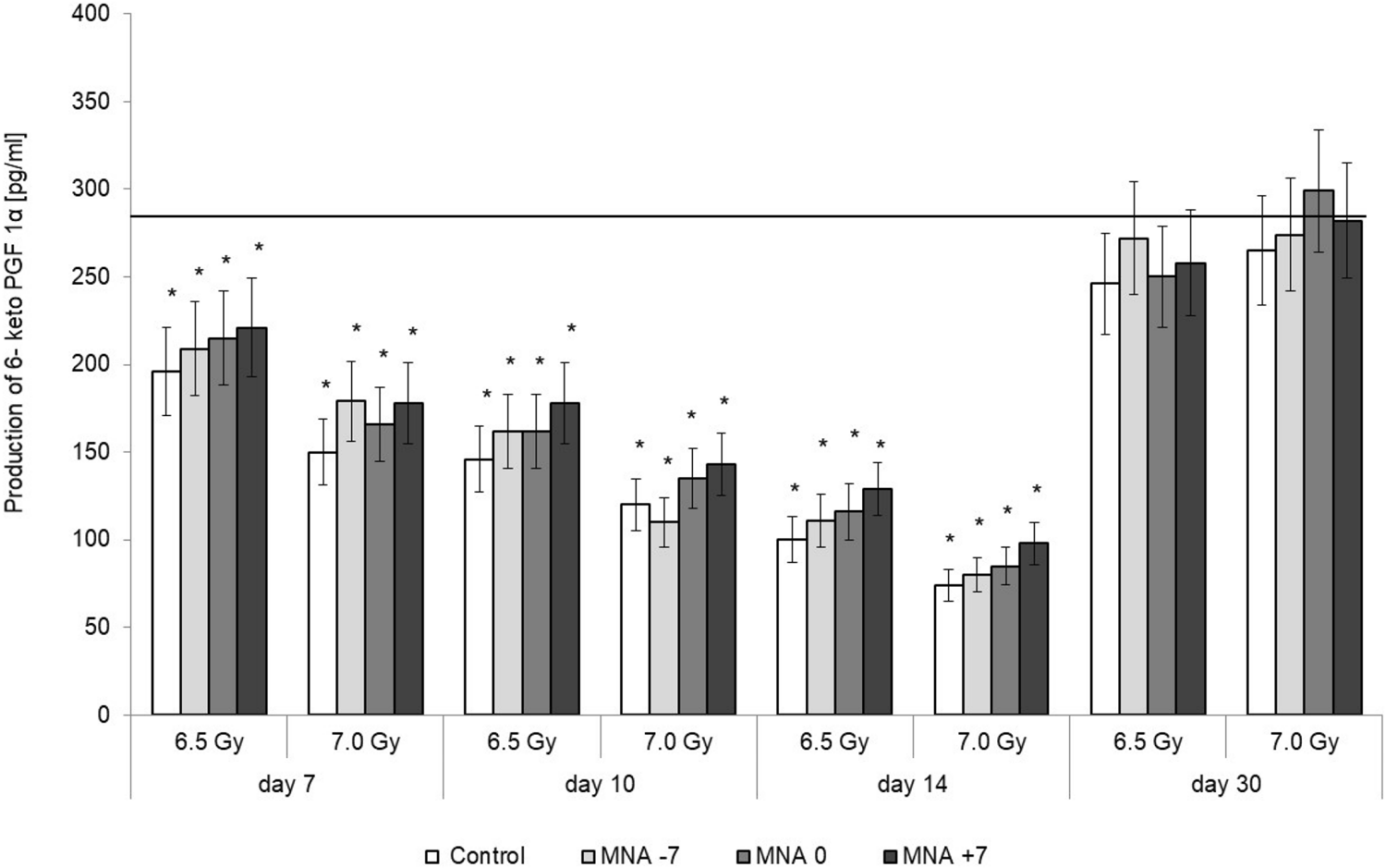
Serum levels of 6-keto PGF1α [pg/ml] in BALB/c mice exposed to WBI at 6.5 or 7.0 Gy γ-rays and fed 1-methylnicotinamide (MNA) in drinking water (100 mg/kg b.m./day) starting 7 days before, on the day of, or 7 days after WBI. Mean values ± SD obtained from experiments conducted on 20 mice per group are presented; Day 7—7th day after WBI at 6.5 or 7.0 Gy γ-rays; Day 10—10th day after WBI at 6.5 or 7.0 Gy γ-rays; Day 14—14th day after WBI at 6.5 or 7.0 Gy γ-rays; Day 30—30th day after WBI at 6.5 or 7.0 Gy γ-rays; 6.5 Gy—mice exposed to WBI at 6.5 Gy γ-rays; 7.0 Gy—mice exposed to WBI at 7.0 Gy γ-rays; Control—mice exposed to WBI at 6.5 or 7.0 Gy γ-rays; MNA-7—mice fed MNA from the 7th days before WBI; MNA0—mice fed MNA from the day of WBI; MNA + 7—mice fed MNA from the 7th day after WBI. The solid line represents the serum level of 6-keto PGF F1α in the sham-irradiated mice. *Indicates statistically significant (*p* < 0.05) difference from the results obtained in the sham-irradiated mice.

In order to assess the possible involvement of the thrombotic vascular reactions in the observed effects, the levels of prostacyclin I_2_ (PGI_2_), quantitated based on the level of 6-keto prostaglandin F1α (6-ketoPGF1α), and thromboxane A2, quantitated based on the level of thromboxane B2 (TXB2), were examined in the plasma of the irradiated mice fed MNA, the seemingly most potent radioprotective/radiomitigative/radioremedial compound in this investigation. As shown in Fig. 7, WBI at 6.5 or 7.0 Gy γ-rays resulted in the significant dose-dependent decrease in the production of PGI_2_ which was noted until the 14th day after the irradiations. However, 2 weeks later the concentration of PGI_2_ almost reverted to the level observed in the sham-exposed, control mice (solid line). Administration of MNA tended to recover the production of PGI_2_, the effect being most pronounced when the animals were given MNA from the 7th day after WBI (Fig. 7). No changes, however, were detected in the production of thromboxane in mice irradiated at LD_30/30_ or LD_50/30_ regardless of whether or not they were fed MNA.

## Discussion

For the present study we used BALB/c mice which compared to e.g., C57BL/6 mice are relatively radiosensitive. This sensitivity as well as cancer proneness of BALB/c mice is associated with inadequate repair of the double-strand breaks induced in these mice by various stress factors^13^. We assumed that if a radiation countermeasure was effective in radiosensitive animals, it would likely be even more efficient in radioresistant ones.

In the study we have demonstrated that some of the tested derivatives of vitamin B_3_ exert radioprotective, radiomitigative, and/or radioremedial effects in the relatively radiosensitive BALB/c mice exposed to 6.5, 7.0, or 7.5 Gy of γ-rays, i.e., at LD_30/30_, LD_50/30_ or LD_80/30_. The effects were assessed with use of the 30-day survival assay as recommended by the FDA for studies of radiation countermeasures in rodents^14^. Of the five tested compounds, one of them (MNA) exerted both radioprotective (i.e., when application of the compound started 7 days before the irradiation and was continued until the animals’ death or the last day of the observation) and radioremedial (when the application started 7 days post irradiation) properties, while NAc showed only radiomitigative (when its application started on the day of irradiation) properties and 1,3 MAP—only radioremedial properties. Notably, MNA was the only compound which upon administration from the 7th day after the irradiation significantly (1.6 to 1.9 times) reduced the mortality rate of mice exposed to all the three doses of γ-rays. It is unclear why application of MNA at earlier times (except for day 7 before the irradiation, but only at 7.5 Gy) was not similarly effective. It can be argued that the efficacy of the ‘remedial’ agent can manifest itself only after, and not before, the ‘recoverable’ alterations have been fully developed. Indeed, since ionizing radiation is likely to inhibit the activity of nicotinamide N-methyltransferase responsible for the production of endogenous MNA from nicotinamide^15,16^ it is likely that the deficit of endogenous MNA develops as late as several days post irradiation and only then supplementation with exogenous MNA can be effective in terms of, e.g., thwarting the ensuing inflammation and ‘cytokine storm’ in tissues. This may also be the likely explanation why MNA was ineffective when its administration started on the day of irradiation, but cannot explain its efficacy when administered from day 7 before the irradiation at 7.5 Gy. This latter observation could be regarded as an ‘artifact’ given that no such effect was detected in mice exposed at 7 and 6.5 Gy. Radioremedial activity was also observed after application of 1,3 MAP, but, surprisingly, the effect was significantly expressed only in mice exposed to the highest dose of γ-rays (Fig. 1). Again, it may be speculated that the effectiveness of the 1,3 MAP could be detected only in mice in which the radiation-induced ‘alterations’ have already been fully expressed and when external supplementation of 1,3 MAP would normalize the radiation-induced profound deficiency of the endogenous level of this compound. Further studies are certainly needed to clarify these uncertainties.

To our knowledge, these results are the first to demonstrate decreased mortality of the lethally irradiated mice following administration of the vitamin B_3_ derivatives beginning as late as 7 days after the irradiation. Indeed, in most of the published reports to date the compounds examined in various experimental models were administrated either before or shortly after exposure to lethal doses of ionizing radiation. In numerous studies a whole spectrum of natural substances including vitamin derivatives have been tested for their radioprotective (i.e., prophylactic), but not radiomitigative or radioremedial properties. For example, survival of CD2F1 mice was significantly enhanced when isomers of vitamin E (α-, γ-, and δ-tocotrienols) were injected subcutaneously (s.c.) 24 h before exposure of the animals to doses ranging from 8.75 to 11.5 Gy of γ-rays^17–19^. Similar effect was demonstrated in BALB/c mice^20^ and Wistar rats^21^ intraperitoneally (i.p.) injected with melatonin, a naturally occurring immune stimulator and free radical scavenger, 1 h or 30 min prior to irradiation at 4 Gy of carbon ions or 10 Gy of γ-rays, respectively. Likewise, the *Mentha* extract given to Swiss mice for three consecutive days before their exposure to WBI with γ-rays at 8 Gy led to the markedly increased 30-day survival of the animals^22^. When Wistar rats were fed Annonaceae, a dried extract from *Xylopia aethiopica*, or vitamin C for 6 weeks before and 8 weeks after WBI at 5 Gy γ-rays the 8-week survival rate of the animals was significantly increased^23^. In another study, injection of Swiss mice with GST-TAT-SOD, a product of the fusion of glutathione-S-transferase (GST), a cell-permeable peptide TAT and superoxide dismutase (SOD), 2 h before the lethal irradiation at 8 Gy of X-rays led to the markedly increased 30-day survival of the animals^24^. Finally, the 30-day survival was significantly increased in the lethally (at 7.5 or 8 Gy of γ-rays) irradiated C3H/HeN mice which were 15 min. earlier s.c. injected with Ex-Rad, a small molecule kinase inhibitor and modifier of the cell cycle in cancer cells^25^.

Another group of the extensively investigated agents are radiomitigators, i.e. substances administered soon after irradiation in expectation to halt the progression of the radiation-induced tissue damage. Among the studied compounds are immunomodulating cytokines^26^. Thus, the significantly enhanced survival of white inbred mice was demonstrated following application of the recombinant IL-1β 10–15 min. after WBI of the animals at 8 or 9 Gy of X-rays^27^. Increased survival of the lethally irradiated mice from various strains was also observed following application of: (a) the *Mycoplasma*-derived lipopeptide ligand for the Toll-like receptor 2/6 (CBLB613) s.c. injected to CD2F1mice either 24 h before or 1 h after the irradiation at 9.2 Gy of γ-rays^28^; (b) a somatostatin analogue SOM230 administered s.c. to CD2F1 mice twice daily from 2 days before or 4 h after WBI at 9 Gy γ-rays and continued for either 14 or 21 days^29^; (c) tetracycline given to C3Hf/Kam mice in five daily i.p. injections starting 24 h after the irradiation at 8 Gy of γ-rays^30^, and (d) a *Saccharomyces cerevisiae*-derived powder containing Zn, Mn, Cu, or Se i.p. injected to C3H mice 30 min before, immediately after, or 10 h post-irradiation at 7.5 Gy of X-rays^31^. To our knowledge, none of the above described or other agents investigated to date have been shown to effectively improve survival of experimental animals irradiated at LD_30/30_, LD_50/30_ or LD_80/30_ when an agent was applied later than 1–2 days after the irradiation.

As expected, our present studies demonstrated that WBI of mice at 6.5–7.5 Gy of γ-rays dramatically and proportionally to the dose reduced the counts of leukocytes, platelets, and erythrocytes in the blood as well as the total numbers of cells in the bone marrow and spleen (Table 1). Even though the animals spontaneously recovered from this depression of haematopoiesis by about 30th day post irradiation, application of any of the tested vitamin B_3_ derivatives did not seem to precipitate the recovery, since no significant increases in the numbers of blood, bone marrow, and spleen cells were noted in the irradiated mice fed these compounds. It is therefore unlikely that stimulation of the haematopoietic system was responsible for the pro-survival activities of these compounds in mice exposed at LD_30/30_, LD_50/30_, or LD_80/30_ of γ-rays. To our knowledge, the only derivative of vitamin B_3_ tested thus far for its haematopoietic effects was nicotinamide. Although NA stimulated polyploidization of megakaryocytes in vitro, regular injections of this compound to C57Bl/6 mice did not result in an increase of the platelet counts in the blood^32–34^. Likewise, as reported by Fu and co-workers, prolonged survival of the lethally irradiated CD2F1 mice induced by application of a naturally occurring analogue of somatostatin (SOM230) was not associated with mitigation of the radiation-induced haematopoietic depression^29^. These results, however, contrast with most of the other data demonstrating that the observed radioprotective effects of the examined compounds were associated with stimulation of haematopoiesis. For example, Yi et al.^4^ assessed haematological parameters in mice exposed to 5.5 Gy γ-rays and demonstrated that a 1-h pre-treatment of the animals with 1,2-propanediol (PPD), a drug solvent and a humectant, significantly elevated the blood counts of erythrocytes, leukocytes, and platelets as estimated between 10 and 30 days post irradiation. Application of various agents of natural origin, such as melatonin, γ- and δ-tocotrienols, the *Mentha piperita* extract, CBLB613, GST-TAT-SOD, and recombinant IL-1β from 24 h before to 6 h post irradiation led to the recovery of haematopoiesis expressed by the elevated numbers of circulating leukocytes, platelets and erythrocytes and the haemoglobin and haematocrit values as well as by the increased spleen mass and bone marrow cellularities in different strains of mice^17,18,20,21,24,27,28,35,36^.

High absorbed doses of ionizing radiation are potent inducers of inflammation^37,38^. This effect has been reflected by our present results demonstrating a significant and prolonged up-regulation of the serum levels of a number of pro-inflammatory cytokines in mice exposed to 6.5 Gy of γ-rays. However, it is possible, that the serum levels of pro-inflammatory cytokines (IL-1 β, IL-6, IL-8, and TNFα) do not reflect the real local levels of these cytokines. When the irradiated mice were given MNA, NAc, or 1,3-MAP from either 7 days before, the day of, or 7 days after WBI the elevated levels of IL-1β, IL-6, IL-8, and TNF-α gradually decreased and by the 30th day post irradiation were, in most cases, close to the baseline level. These results corroborate our and other authors’ earlier demonstrations that application of the MNA can down-regulate pro-inflammatory cytokines^9,12^. The anti-inflammatory activity of MNA may explain our present finding that this compound was most effective when applied from the 7th day post irradiation. It is possible that ionizing radiation inhibits the activity of nicotinamide N-methyltransferase (NNMT) responsible for metabolism of NA to MNA and reduces thereby the endogenous level of the latter. Thus, supplementation of this compound after the irradiation is likely to eliminate the deficit of endogenous MNA which may act as a vasoprotective mediator. Indeed, the upregulated NNMT/MNA ratio may represent a compensatory mechanism^39,40^ and MNA supplementation may mimic this response. It remains to be established, however, what is the effect of WBI on the plasma concentration of MNA. Altogether, the obtained results suggest that attenuation of inflammation by MNA, NAc, or 1,3-MAP could at least partially explain the pro-survival effects of these compounds. Similar findings were recently reported by only two groups of investigators who, however, tested different substances: the first one showed that the radiation-induced elevation of IL-6 and TNF-α in the blood could be reversed by i.p. injection of melatonin 1 h before WBI of BALB/c mice with carbon ions at total dose of 4 Gy^20^ and the second demonstrated that administration of *Podophyllotoxin* and the Rutin formulation G-003 M prepared from a plant *Podophyllum hexandrum* 1 h prior to exposure of C57BL/6 mice to the lethal doses of γ-rays mitigated the radiation-induced inflammation in the lungs and intestine mediated by IL-6, TNF-α, and TNF-γ^41^.

Radiogenic organ pathologies may also be associated with the impaired function of endothelium. It seems that a key element in the development of radiation-induced endothelial dysfunction is the increased generation of free oxygen species accompanied by the activation of inflammatory and thrombotic processes which contribute to the impairment of blood supply to tissues and, in extreme cases, to the multi-organ failure^42–47^. In view of the described anti-thrombotic properties of MNA likely to be mediated by a prostacyclin (PGI_2_)-dependent mechanism^6^ it is possible that such properties may be involved in the survival-enhancing effects of this vitamin B_3_ derivative, the most potent radioprotective/radiomitigative/radioremedial compound in this investigation. Indeed, in accord with the previous demonstrations of the stimulated secretion of PGI_2_ by MNA, 1,3-MAP, and NAc^6^ we demonstrated in the present study that application of MNA to mice either from day 7 before, the day of, or day 7 after exposure to 6.5 or 7.0 Gy of γ-rays tended to up-grade the radiation-induced depressed level of PGI_2_ in the serum as manifested between the 7th and the 14th day after the irradiation (Fig. 7). Hence, the PGI_2_-mediated anti-thrombotic and/or vasoprotective effects could also be considered as possible underlying mechanisms of the survival-enhancing activity of MNA in severely irradiated mice.

In conclusion, in the present study we demonstrate for the first time, that the selected derivatives of vitamin B_3_ significantly increase survival of the lethally irradiated BALB/c mice when application of these compounds starts a week before (in case of MNA), on the day of (NAc), or—most notably—as late as a week after (MNA, 1,3-MAP) the irradiation. In these mice stimulation of haematopoiesis does not seem to be the underlying mechanisms of the enhanced survival. However, the significant reduction of the levels of the radiation-induced pro-inflammatory cytokines and the slightly enhanced production of PGI_2_ suggests that anti-inflammatory, anti-thrombotic, and vasoprotective activities of the examined compounds, as described previously^5,6,11^, may be involved. The obtained results will likely contribute to verification of the prevailing belief that the currently known ‘radioprotectors’ are either totally ineffective when applied after the irradiation or practically useless owing to the associated toxicity. Most importantly, the results of our studies should guide further investigations into the endothelium-targeted vasoprotective therapeutics, that could foster the development of effective treatment of radiation injuries when it starts several hours or days post-exposure and can be applied to patients undergoing radiotherapy as well as in victims of radiation accidents and criminal acts in which radiological and/or nuclear weapons have been used.

## Materials and methods

### Animals

Male BALB/c mice were obtained from the Nofer Institute of Occupational Medicine, Lodz, Poland, and at 6–8 weeks of age were used for the experiments. All the animals were maintained under specific pathogen-free conditions. During the experiments, the mice were provided with a natural daily cycle (12-h photoperiod), had access to food and water ad libitum and were housed in a Modular Animal Caging System—MACS Mobile Units (Alternative Design, Siloam Springs, USA). The living conditions and health of the animals were monitored on a regular basis by a veterinarian. The mice were randomly assigned to the experimental groups. Random numbers were generated using the standard = RAND() function in Microsoft Excel. The investigations were carried out by permission of the II Local Ethical Committee for Experimentation on Animals at the National Medicines Institute in Warsaw, Poland (permission No. 78/2009). We confirm that all the experiments were performed in accordance with all the relevant guidelines and regulations. We also confirm that we complied with ARRIVE guidelines.

### Irradiation

The mice were exposed to whole-body irradiation (WBI) from a ^60^Co source at 7.2 Gy/h mean dose rate to obtain the absorbed doses of 6.5, 7.0 or 7.5 Gy per mouse. During the irradiations the animals were placed in a ventilated container and positioned along the axis of the radiation beam. All the exposures were performed at the Military Institute of Chemistry and Radiometry, Warsaw, Poland. The absorbed doses were verified using thermoluminescent dosimeters (RADCARD, Cracow, Poland) implanted s.c. in the middle abdominal region of an animal.

### Examined derivatives of vitamin B_***3***_

For the experiments the following compounds were used: nicotinic acid (NAc)—a form of vitamin B_3_, nicotinamide (NA)—an amide of nicotinic acid, also a form of vitamin B_3_, 1-methylnicotinamide (MNA)—a primary metabolite of NA, and 1-methyl-3-acetylpyridine (1,3-MAP)—an analogue of MNA with an acetyl group in the 3-position of the pyridine ring. All these compounds were obtained from the Institute of Applied Radiation Chemistry, the Lodz University of Technology, Lodz, Poland.

### Application of the examined compounds

The vitamin B_3_ derivatives were dissolved in drinking water and given to the animals at concentrations assuring their daily consumption at approx. 100 mg/kg body mass (b.m.). The dose used in this study was based on previous experience in using these compounds in different studies^6,11,40^. The water with dissolved compounds was replaced daily. The amount of the water drunk by the mice per day was assessed during the pilot studies. Applications of the compounds started 7 days before, on the day of, or 7 days after WBI and were continued until the animals’ death or the last day of the observation. Control animals were given clean water containing no additives.

### Survival assay

Survival of the animals was assessed over 30 days after WBI by daily inspection of the cages with mice from the experimental groups consisting of 24 animals each (including the pilot study) and the results are presented in percent of the dead mice per group. Mice were monitored using standard criteria for humane euthanasia as an endpoint.

### Spleen and bone marrow cellularity

Mice were sacrificed with Isoflurane using the euthanasia induction box (Witko, Lodz, Poland) and spleens and bone marrow were removed from the abdominal cavity and collected from the femurs, respectively. The spleens were minced and the obtained cells from the spleens and bone marrow were suspended in PBS (BioMed-LUBLIN, Lublin, Poland). The resulting single-cell suspensions were quantitated in a mammalian cell counter NucleoCounter NC-100 (ChemoMetec, Allerød, Denmark). Each experimental group consisted of 20 animals. The days of the estimations (7, 10, 14, and 30 days post-irradiation) were chosen based on the literature data^4^ and the survival curves obtained in the present study. From each experimental group four animals were picked up at random for the secondary endpoint analyses (that is why the initial number of the animals per group was as ‘large’ as twenty). The culled animals were ‘chosen’ blindly, i.e., without regard for their behavior or general condition.

### Peripheral blood cell counts

Mice were anesthetized with Isoflurane using of the anaesthesia induction box (Witko, Lodz, Poland) and blood samples were collected by heart puncture of anesthetized mice and blood cell counts were estimated in a haematological analyzer Mysthic 18 (Cormay, Lomianki, Poland). Each experimental group consisted of 20 animals. The days of the assessment (7, 10, 14, and 30 days post-irradiation) were chosen based on the literature data^4^ and the survival curves obtained in the present study*.* From each experimental group four animals were picked up at random for the secondary endpoint analyses (that is why the initial number of the animals per group was as ‘large’ as twenty). The culled animals were ‘chosen’ blindly, i.e., without regard for their behavior or general condition.

### Production of cytokines, prostacyclin, and thromboxane

Serum and plasma samples were collected from peripheral blood obtained by heart puncture of anesthetized mice and then frozen at − 70 °C. Defrosted serum/plasma was assayed with the ELISA using a microplate spectrophotometer Epoch (BioTek Instruments, Inc., Vermont, USA) and a microplate strip washer ELx50 (BioTek Instruments, Inc., Vermont, USA). The obtained data were analyzed with the use of a reader software Gen5 2.0 (BioTek Instruments, Inc., Vermont, USA). The levels of cytokines were determined using the respective tests: IL-1β—Quantikine ELISA Mouse IL-1β/IL-1F2 Immunoassay (R&D Systems, Inc., Minneapolis, USA), IL-6—Quantikine ELISA Mouse IL-6 Immunoassay (R&D Systems, Inc., Minneapolis, USA), IL-8—Quantikine ELISA Mouse IL-8 Immunoassay (R&D Systems, Inc., Minneapolis, USA), TNF-α—Quantikine ELISA Mouse TNF-α Immunoassay (R&D Systems, Inc., Minneapolis, USA). The level of prostacyclin (PGI_2_) was assessed using the 6-keto Prostaglandin F1α EIA Kit (Cayman Chemical Company, Michigan, USA), and the level of thromboxane—using the Thromboxane B2 Express Kit (Cayman Chemical Company, Michigan, USA).

### Statistical analysis

To compare survival distributions between the groups, the log rank (Mantel-Cox) test was used and *p* values less than 0.05 were regarded as significant.

For statistical analysis of the inter-group differences in spleen and bone marrow cellularity, peripheral blood cell counts or levels of cytokines, PGI_2_, and thromboxane the Mann–Whitney U test for non-parametric trials was used with *p* values less than 0.05 regarded as significant.

## Supplementary Information

Supplementary Information 1.

Supplementary Information 2.

Supplementary Information 3.

Supplementary Information 4.
