# Supplementary material for: A derivative of vitamin B3 applied several days after exposure reduces lethality of severely irradiated mice
Source: Sci Rep. 2021 Apr 12;11:7922. doi: 10.1038/s41598-021-86870-3 (PMC8041812; doi:10.1038/s41598-021-86870-3)

Supplementary Fig.1. Condition of BALB/c mice at the end of the 30-day survival assay after their WBI at7.5 Gy γ-rays and feeding with MNA from day 7 post-WBI.


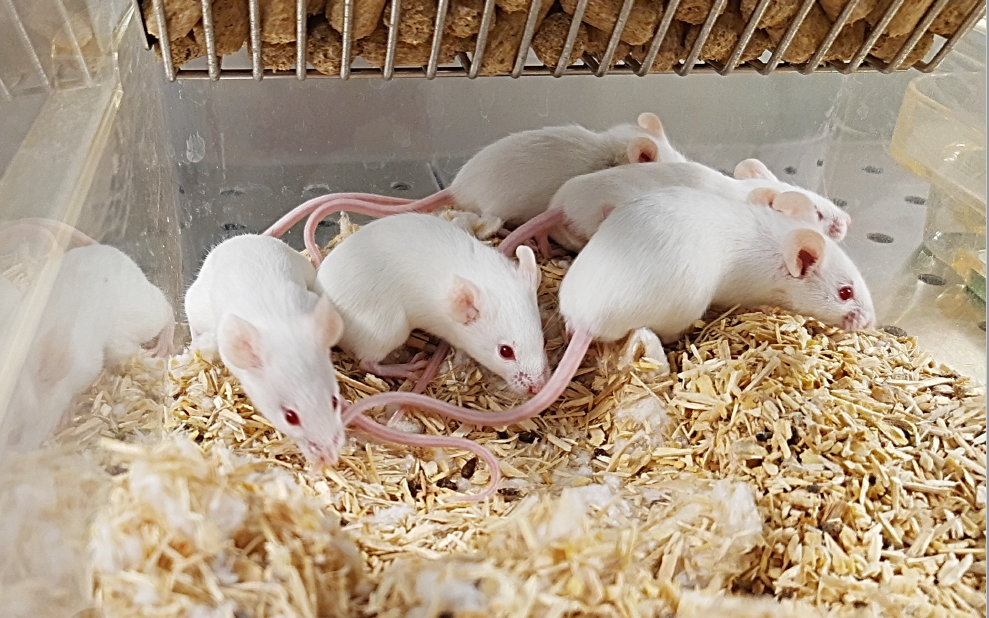

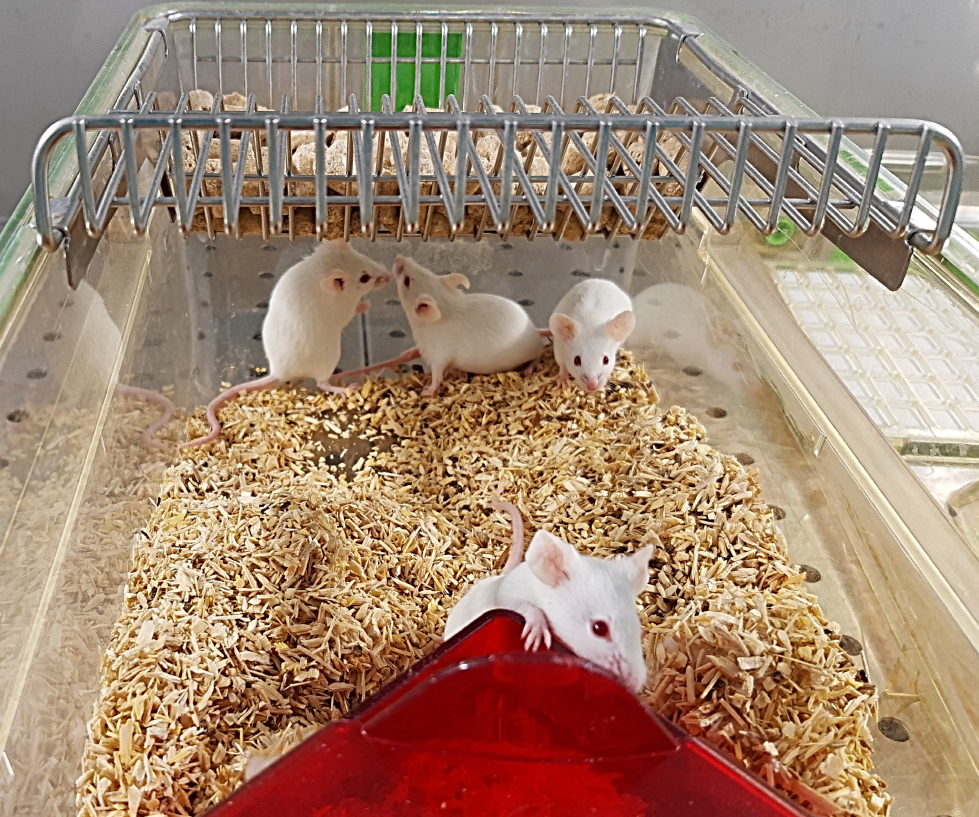

Supplement: Supplementary file 1 — Supplementary Information 1. [file 41598_2021_86870_MOESM1_ESM.doc]
