# Supplementary material for: A derivative of vitamin B3 applied several days after exposure reduces lethality of severely irradiated mice
Source: Sci Rep. 2021 Apr 12;11:7922. doi: 10.1038/s41598-021-86870-3 (PMC8041812; doi:10.1038/s41598-021-86870-3)
Supplement: Supplementary file 2 — Supplementary Information 2. [file 41598_2021_86870_MOESM2_ESM.doc]

Supplementary Fig.2. Serum levels of interleukin 1β (IL-1beta), interleukin 6 (IL-6), interleukin 8 (IL8), and tumour necrosis factor α (TNF-alfa) [pg/ml] in BALB/c mice exposed to WBI at 6.5 Gy γ-rays and fed nicotinamide (NA) in drinking water (100 mg/kg b.m./day)


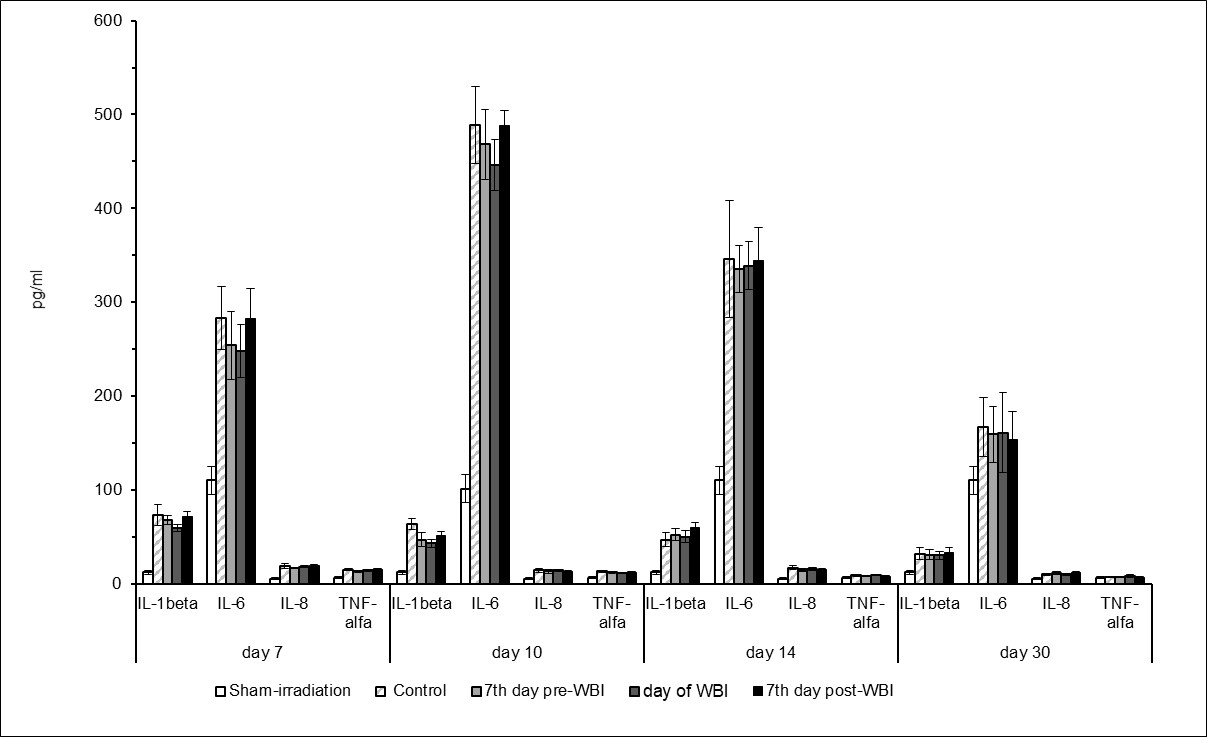


Mean values ± SD obtained from experiments conducted on 20 mice per group are presented. Day 7 – 7th day after WBI at 6.5 Gy γ-rays; Day 10 – 10th day after WBI at 6.5 Gy γ-rays; Day 14 – 14th day after WBI at 6.5 Gy γ-rays; Day 30 – 30th day after WBI at 6.5 Gy γ-rays; Sham-irradiation – sham-irradiated mice; Control – mice exposed to WBI at 6.5 Gy γ-rays without any other treatment; 7th day pre-WBI – mice exposed to WBI at 6.5 Gy γ-rays and NA from the 7th day before WBI; day of WBI – mice exposed to WBI at 6.5 Gy γ-rays and fed NA from the day of WBI; 7th day post-WBI – mice exposed to WBI at 6.5 Gy γ-rays and fed NA from the 7th day after WBI.
