# Supplementary material for: A derivative of vitamin B3 applied several days after exposure reduces lethality of severely irradiated mice
Source: Sci Rep. 2021 Apr 12;11:7922. doi: 10.1038/s41598-021-86870-3 (PMC8041812; doi:10.1038/s41598-021-86870-3)
Supplement: Supplementary file 3 — Supplementary Information 3. [file 41598_2021_86870_MOESM3_ESM.doc]

Supplementary Table 1. Increases in body mass of BALB/c mice at the end of the 30-day survival assay after their exposure to WBI at 6.5, 7.0, and 7.5 Gy γ-rays.

| **Groups** | | | **Body mass [g]** | | |
| --- | --- | --- | --- | --- | --- |
|  |  |  | **Day 0** | **Day 30** | **Day 30 – Day 0** |
| **0 Gy - (control)** | | | 23.4 ± 1.7 | 27.5 ± 1.6 | 4.1 |
| **6.5 Gy** | NAc | 7th day pre-WBI | 22.8 ± 1.5 | 27.1 ± 1.9 | 4.3 |
|  |  | day of WBI | 23.0 ± 1.5 | 27.2 ± 1.8 | 4.2 |
|  |  | 7th day post-WBI | 22.7 ± 2.2 | 28.8 ± 2.6 | 6.2 |
|  | NA | 7th day pre-WBI | 24.3 ± 1.1 | 27.4 ± 1.3 | 3.1 |
|  |  | day of WBI | 22.5 ± 1.5 | 26.7 ± 1.8 | 4.2 |
|  |  | 7th day post-WBI | 24.3 ± 1.4 | 28.2 ± 1.7 | 3.9 |
|  | MNA | 7th day pre-WBI | 22.4 ± 1.5 | 26.7 ± 1.8 | 4.3 |
|  |  | day of WBI | 23.6 ±1.2 | 26.8 ± 1.4 | 3.2 |
|  |  | 7th day post-WBI | 23.7 ± 1.7 | 28.4 ± 2.0 | 4.7 |
|  | 1,3-MAP | 7th day pre-WBI | 23.4 ± 1.3 | 26.9 ± 1.5 | 3.5 |
|  |  | day of WBI | 22.2 ± 1.4 | 26.1 ± 1.7 | 3.9 |
|  |  | 7th day post-WBI | 22.7 ± 1.6 | 27.9 ± 2.2 | 5.2 |
| **7.0 Gy** | NAc | 7th day pre-WBI | 23.7 ± 1.0 | 26.5 ± 1.2 | 2.8 |
|  |  | day of WBI | 20.2 ± 1.4 | 24.1 ± 1.7 | 3.9 |
|  |  | 7th day post-WBI | 22.4 ± 1.0 | 27.4 ± 2.2 | 5.0 |
|  | NA | 7th day pre-WBI | 24.2 ± 1.8 | 27.1 ± 1.2 | 2.9 |
|  |  | day of WBI | 26.7 ± 1.1 | 29.7 ± 1.3 | 3.0 |
|  |  | 7th day post-WBI | 24.3 ± 1.4 | 28.2 ± 1.7 | 3.9 |
|  | MNA | 7th day pre-WBI | 22.4 ± 1.6 | 26.9 ± 1.9 | 4.5 |
|  |  | day of WBI | 21.7 ± 1.6 | 26.1 ± 1.9 | 4.4 |
|  |  | 7th day post-WBI | 23.7 ± 1.0 | 26.4 ± 1.2 | 2.7 |
|  | 1,3-MAP | 7th day pre-WBI | 22.9 ± 1.5 | 27.2 ± 1.8 | 4.3 |
|  |  | day of WBI | 24.3 ± 1.2 | 27.7 ± 1.5 | 3.4 |
|  |  | 7th day post-WBI | 23.1 ± 1.4 | 27.1 ± 1.7 | 4.0 |
| **7.5 Gy** | NAc | 7th day pre-WBI | 19.6 ± 1.8 | 24.5 ± 2.1 | 4.9 |
|  |  | day of WBI | 21.9 ± 1.4 | 25.7 ± 1.6 | 3.8 |
|  |  | 7th day post-WBI | 23.9 ± 1.2 | 27.3 ± 1.5 | 3.4 |
|  | NA | 7th day pre-WBI | 23.5 ± 1.3 | 27.1 ± 1.5 | 3.6 |
|  |  | day of WBI | 24.2 ± 1.6 | 28.8 ± 2.0 | 4.6 |
|  |  | 7th day post-WBI | 25.5 ± 1.0 | 28.2 ± 1.2 | 2.7 |
|  | MNA | 7th day pre-WBI | 22.4 ± 1.2 | 25.8 ± 1.5 | 3.4 |
|  |  | day of WBI | 23.9 ± 1.4 | 27.9 ± 1.7 | 4.0 |
|  |  | 7th day post-WBI | 22.2 ± 1.3 | 25.9 ± 1.6 | 3.7 |
|  | 1,3-MAP | 7th day pre-WBI | 22.9 ± 1.8 | 27.8 ± 2.1 | 4.9 |
|  |  | day of WBI | 22.2 ± 1.9 | 27.6 ± 2.3 | 5.4 |
|  |  | 7th day post-WBI | 23.2 ± 1.4 | 27.0 ± 1.6 | 3.8 |

Mean values ± SD are presented. 0 Gy (control) – mice sham-exposed to X γ -rays; 6.5 Gy – mice irradiated at 6.0 Gy of γ -rays; 7.0 Gy – mice irradiated at 6.0 Gy of γ -rays; 7.5 Gy – mice irradiated at 6.0 Gy of γ -rays; Day 0 – body mass at the beginning of the 30-day survival assay; Day 30 – body mass at the end of the 30-day survival assay; Day 30 – Day 0 – the difference between body mass at the end and the beginning of the 30-day survival assay; NAc – mice fed nicotinic acid; NA – mice fed nicotinamide; MNA – mice fed 1-methylnicotinamide; 1,3-MAP – mice fed 1-methyl-3-acetylpyridine; 7th day pre-WBI – mice exposed to WBI at 6.5, 7.0, or 7.5 Gy γ-rays and fed the vitamin B3 derivatives from the 7th day before WBI; day of WBI – mice exposed to WBI at 6.5, 7.0, or 7.5 Gy γ-rays and fed the vitamin B3 derivatives from the day of WBI; 7th day post-WBI – mice exposed to WBI at 6.5, 7.0, or 7.5 Gy γ-rays and fed the vitamin B3 derivatives from the 7th day after WBI.
