# Supplementary material for: A derivative of vitamin B3 applied several days after exposure reduces lethality of severely irradiated mice
Source: Sci Rep. 2021 Apr 12;11:7922. doi: 10.1038/s41598-021-86870-3 (PMC8041812; doi:10.1038/s41598-021-86870-3)
Supplement: Supplementary file 4 — Supplementary Information 4. [file 41598_2021_86870_MOESM4_ESM.docx]

Supplementary Table 2. Bone marrow, spleen, and blood parameters in BALB/c exposed to WBI at 6.5, 7.0 or 7.5 Gy γ-rays and fed: 1-methylnicotinamide (MNA), nicotinic acid (NAc), 1-methyl-3-acetylpyridine (1,3-MAP) or nicotinamide (NA) in drinking water (100 mg/kg b.m./day)

######

######

######


Mean values ± SD obtained from experiments conducted on 20 mice per group are presented. BM – bone marrow cells, Spleen – spleen cells, WBC – white blood cells; PLT – platelets; RBC – red blood cells; HTC- haematocrit; HGB - haemoglobin; 6.5 Gy – mice exposed to WBI at 6.5 Gy γ-rays; 7.0 Gy – mice exposed to WBI at 7.0 Gy γ-rays; 7.5 Gy – mice exposed to WBI at 7.5 Gy γ-rays; no treated - mice only exposed to WBI at 6.5, 7.0, or 7.5 Gy γ-rays; 7th day pre-WBI – mice exposed to WBI at 6.5, 7.0, or 7.5 Gy γ-rays and fed the vitamin B_3_ derivatives from the 7th day before WBI; day of WBI – mice exposed to WBI at 6.5, 7.0, or 7.5 Gy γ-rays and fed the vitamin B_3_ derivatives from the day of WBI; 7th day post-WBI – mice exposed to WBI at 6.5, 7.0, or 7.5 Gy γ-rays and fed the vitamin B_3_ derivatives from the 7th day after WBI; Day 7 – 7th day after WBI at 6.5, 7.0 or 7.5 Gy γ-rays; Day 10 – 10th day after WBI at 6.5, 7.0 or 7.5 Gy γ-rays; Day 14 – 14th day after WBI at 6.5, 7.0 or 7.5 Gy γ-rays; Day 30 – 30th day after WBI at 6.5, 7.0 or 7.5 Gy γ-rays.
